# Health resorts as gateways for regional, standardised, sports club based exercise programmes to increase the weekly time of moderate- to vigorous-intensity physical activity: study protocol

**DOI:** 10.1186/s12889-015-2581-9

**Published:** 2015-12-21

**Authors:** Christian Lackinger, Albert Strehn, Thomas Ernst Dorner, Josef Niebauer, Sylvia Titze

**Affiliations:** Department of Health Promotion and Prevention, SPORTUNION Österreich, Falkestrasse 1, 1010 Vienna, Austria; Competence Center Health Promotion, SVA, Osterwiese 2, 7000 Eisenstadt, Austria; Institute of Social Medicine, Centre for Public Health, Medical University of Vienna, Kinderspitalgasse 15/1, 1090 Vienna, Austria; Institut of Sports Medicine, Prevention and Rehabilitation; Paracelsus Medizinische Privatuniversität Salzburg, Lindhofstr. 20, 5020 Salzburg, Austria; Institute of Sports Science, University of Graz, Mozartgasse 14, 8101 Graz, Austria

**Keywords:** Health behaviour change, Community-based exercise programme, Sports clubs, Health resorts, Adults

## Abstract

**Background:**

More than 10 % (approximately 60,000) of the adult population in Styria, a federal state in the south of Austria, is granted a residential stay in a health resort each year. The target group for these stays is the general population aged between 30 and 65 years with minor symptoms such as risk factors for cardio-metabolic diseases. Stays are financed by health insurance companies and last up to three weeks. The treatment during the stays consists of exercise and nutritional intervention as well as psychological support when needed. However, because of the absence of regional programmes linked with the residential stay, the sustainability of the interventions is questionable.

**Methods/Design:**

This prospective, controlled, multicentre, open-label study will compare two groups. Participants will be included in the study if they live in any of eight predefined Styrian regions and do not meet the minimal WHO physical activity guidelines. Those allocated to the intervention group will receive a voucher for 12 regional, standardised, sports club based exercise sessions. The members of the control group will come from different but matched Styrian regions and will receive an informative written brochure. The primary outcome will be the weekly level of health-enhancing physical activity, which will be objectively measured with an accelerometer and supplemented by an activity log book. Together with potential determinants of physical activity it will be assessed before, 10 weeks after and 12 months after the residential stay. Additionally, psychosocial determinants will be assessed by questionnaire and fitness (cardiorespiratory fitness, handgrip, balance) will be measured. In addition to the changes in measurable parameters, processes will be evaluated to learn about the facilitators and barriers of the implementation of the programme.

**Discussion:**

It is known that during the residential stay, participants are receptive to new opportunities supporting health behaviour change, but that these measures are not sustained after discharge. The structured cooperation between the health sector that has to inform the participants and the sports sector that provides the wide network of standardised programmes is the strength of the study, but at the same time a challenge.

**Trial registration:**

ClinicalTrials.gov (Identifier: NCT02552134; date of registration: 15 September 2015)

## Background

Physical activity is an important predictor for health. According to the national and international guidelines, a minimum of 150 min of aerobic moderate-intensity physical activity or ≥75 min of vigorous-intensity physical activity, or an equal combination of both, is needed to gain substantial health benefits. In addition to aerobic activities, muscle-strengthening activities that involve all major muscle groups should be performed regularly, at least twice a week [1–3].

In Austria, only a minority of the adult population meet the physical activity guidelines [4, 5].

In Styria, a federal state of Austria, there is a population of 597,033 adults within the age range 30 to 65 years [6]. Out of these, about 60,000 persons are assigned to attend a residential stay at a health resort each year. The approval and payment for these stays is the responsibility of health insurance companies. The target group for these stays at health resorts is people who are basically healthy but show one or more health risk factors. The stays last at least one week, but most last up to three weeks. During the stays, comprehensive lifestyle interventions are provided taking physical activity, nutrition and mental health into account. The stays at the health resorts are different to stationary rehabilitation, where a disease-related treatment is obligatory. However, most of the stationary interventions – preventive as well as rehabilitative – lack sustainability [7]. As a result, the major problem is the missing link between the health resorts and – in the case of physical activity – regional health-enhancing physical activity programmes run by trained personnel [8].

Based on experience in a previous nationwide feasibility study [9, 10], a working group was established representing health insurance companies, national sports umbrella organisations and sports science professionals in the federal state of Styria. The working group established the so-called “HEPA-Styria” (**H**ealth-**E**nhancing **P**hysical **A**ctivity) project linking the health and sports sectors [11]. The project focuses on two fields of activity: 1) during a residential stay at a health resort, the provision of gateways for regional HEPA programmes by the establishment of minimal counselling; and 2) the organisation of regional, standardised, sports club based exercise programmes. As a result of this intervention, it is aimed that people who participate in the regional exercise programmes will be more likely to increase their health-enhancing physical activity than those who do not participate in the exercise programmes. Additionally, determinants of regular physical activity, as well as selected health outcomes, are expected to improve within the intervention group.

## Methods/Design

### Overview

The proposed study is designed as a prospective, controlled, multicentric open-label study that will take place in the federal state of Styria, Austria. Austrian health resorts (*n* = 149) who accommodate patients from Styria will be involved. Based on the communities people come from, the study participants will be divided into an intervention group and a control group. Eight different communities in the federal state of Styria have been selected. Four communities will provide regional, standardised, sports club based exercise programmes and four matched communities will not offer these programmes in the near future. In short, the process will be as follows. People from the eight communities will be approached before the residential stay and asked whether they agree to fill in a questionnaire and participate in a seven-day physical activity measurement programme. Those who agree and do not meet the WHO physical activity guidelines will be included in the study [3]. During the residential stay, members of the intervention group will receive information about the regional, standardised, sports club based exercise programmes in their close living environment. Members of the control group will receive a brochure about physical activity.

The study was approved by the local ethical committee (University of Graz, EK-NR 86-2014/15) and will be conducted according to the principles of the Declaration of Helsinki. Furthermore, the protocol was registered at ClinicalTrials.gov (identifier: NCT02552134).

### Study objectives

The primary outcome of this study will be the weekly level of accelerometer-determined health-enhancing physical activity, measured as minutes of moderate- to vigorous-intensity physical activity (MVPA) [12, 13]. The particular research questions are: 1) How many of the eligible people in the intervention group will participate in the regional, standardised, sports club based exercise programmes after the residential stay? 2) Are those who participate in the exercise programmes more likely to increase their physical activity behaviour than those who do not participate in the exercise programmes?

### Eligibility and recruitment

One hundred and ninety four adults between 30 and 65 years will take part in this study. The main inclusion criteria are: 1) a stay in an Austrian health resort; 2) insufficient levels of physical activity before the stay in the health resort (determined with accelerometers); and 3) resident in one of the eight selected Styrian communities. The main exclusion criteria are: 1) any contraindication concerning physical activity advised by a medical doctor; and 2) pregnant women. Further inclusion and exclusion criteria are presented in Table 1.

**Table 1:** Inclusion and exclusion criteria

Inclusion criteria	
Age ≥30 years and	
Age ≤65 years	
Austrian physical activity recommendations for substantial health benefits are not reached. (Weekly <150 minutes of aerobic moderate-intensity physical activity, or <75 minutes of aerobic vigorous-intensity physical activity, or an equal combination of both, and/or muscle-strengthening activities at least twice a week are not realised.)	
Residential stay at a health resort	
Physical activity in the patient’s responsibility is recommended	
Systolic blood pressure at rest ≤90 mm/Hg	
Diastolic blood pressure at rest ≤140 mm/Hg	
Asymptomatic ECG at rest	
Exclusion criteria	
More than 10 % planned or unplanned weight loss/weight gain during the last six months	
Pregnancy	
Indication for residential or outpatient rehabilitation	
Untreated coronary heart disease	
Untreated micro- or macro-vascular artery disease	

In detail: four Styrian communities were selected according to the postal code as intervention regions because they have well-developed sports club networks. In these urban and rural communities, regional, standardised, sports club based exercise programmes will be provided twice a week. Because the number of inhabitants of three of the communities is relatively small, we decided to selected similar communities as controls and not to randomly select people of the same area for the intervention and control groups. The controls live in four different regions that are similar with regard to size, urban or rural alignment and sports club networks. As soon as the inhabitants of the eight communities have received approval from their health insurance companies for the residential stay in a health resort, they will be contacted and asked whether they agree to the measurement of their physical activity behaviour before the residential stay, and they will also be asked to fill in a questionnaire. If they are willing to take part in the activity analysis, they will have to return a form giving consent to the procedure and providing the assumed start day of the residential stay. Those who do not meet the WHO physical activity guidelines will be eligible for the study and will be asked during their residential stay whether they agree to participate in the study.

### Intervention

During the residential stay in the health resort, members of the intervention group who do not meet the physical activity guidelines based on the accelerometer data will receive a so-called “starter package”. During the stay at the health resort, people who took part in the prior physical activity measurement programme will be informed about the regional programmes if they do not meet the physical activity guidelines.

This starter package will include information about the project, an informed consent form, as well as a voucher for 12 regional, standardised, sports club based exercise sessions which can be attended immediately after the stay. The costs for these 12 sessions will be covered by the project funds so that participants need not pay any fee. Ideally, the beginning of the regional, standardised, exercise programme should be scheduled during the residential stay. “Standardised” means that the instructors will receive an obligatory 18-h training programme, and the sessions will have a defined structure with the following quality criteria [14]:

- Number of exercise sessions per week: two sessions.
- Duration of each single session: 90 min.
- Maximum number of people in a training group: 12 people.
- Cardiovascular exercise during a single session: ≥40 min of moderate- to vigorous-intensity aerobic activities.
- Muscle-strengthening activities during one session: ≥30 min; ≥2 sets of ≥6 different muscle-strengthening activities.
- Coordination and flexibility: will not be performed as a distinct part but it will be integrated in the cardiovascular and muscle-strengthening sections of the programme.
- Feedback on individual physical activity goals: as a supplement to the standardised exercise programme, individual “physical activity homework” will be prescribed to ensure that the physical activity recommendations can be realised. Furthermore, participants will be encouraged to support each other by identifying helpful behaviour change strategies and applying social support. Ten minutes will be reserved for this at the beginning and end of each session.

The advanced job training will include lectures and a manual with 12 different predefined sessions. Furthermore, health aspects of physical activity will be introduced, as well as extended communications training.

The provision of regional, standardised, sports club based exercise programmes in the close neighbourhood should facilitate the transfer from the residential stay to a regional sports club. After the first 12 sessions, participants will be able to continue the standardised programme as a regular member of the local sports club. The annual fee will be €160 per semester.

### Control group

During the stay in the health resort, members of the control group will be encouraged to be active in the future. They will receive the brochure entitled “Physical Activity: Health for all” (Bewegung: Gesundheit für alle http://www.fgoe.org/presse-publikationen/downloads/broschueren-folder/bewegungsbroschure-pdf-475-kb/2013-12-06.3369959292) [15]. The brochure covers the following topics:

- Effects of physical activity.
- How to become active and how to stay active.
- Fitness checks.
- Components of health-enhancing physical activity.
- Body weight.
- Planning your activities.

### Analysis of sub-groups

At the 12-months follow-up, the intervention group will be divided into sub-groups for further analysis:

- Sub-group I: participants who attended ≥75 % of the 12 exercise sessions of the standardised programme during the last 12 months.
- Sub-group II: participants who attended ≥25 % and <75 % of the 12 exercise sessions of the standardised programme during the last 12 months.
- Participants who did not attend 25 % of the 12 exercise sessions of the standardised programme.

The sub-group analysis is necessary because we aim to determine if regular long-term participation leads to different levels of physical activity compared to non-regular participation. Another hypothesis is that finishing the initial 12-session programme is the most powerful predictor for a high level of MVPA. Thus, the sample size calculation is based on between-group differences in MVPA.

### Sample size calculation

For the sample size calculation, the difference in minutes of MVPA measured in bouts ≥10 min is considered as a suitable parameter for health-enhancing physical activity [16]. According to several studies, MVPA is an appropriate parameter for that purpose [17, 18]. Jung et al. [16] evaluated the changes in MVPA after a supervised exercise intervention in a similar cohort (male and females, aged 51(10)) years suffering from prediabetes). After the intervention, MVPA was changed by 44.5 (72.2) minutes. Due to the fact that the intervention-induced changes in our study are supposed to be similar, we considered the changes in MVPA of the above-mentioned study to calculate the sample size in our study.

Given a clinically relevant difference of 44.5 min of MVPA between the intervention and control groups, a standard deviation of 72.2 MVPA of the differences and a two-sided significance level of 0.05, a sample size of *n* = 42 per group is needed to reach a statistical power of 80 %. Since drop-outs and those lost to follow-up may have an inestimable effect on the assumed standard deviation of the differences, the sample size will be increased to *n* = 48 persons per group. Expected value μ1 (intervention) = 84.1 MVPA, μ2 (control) = 39.6 MVPA. Calculations were undertaken via: http://www.clinical-trials.de/de/Werkzeuge/werkzeuge.html

### Logic model

**Fig. 1:**
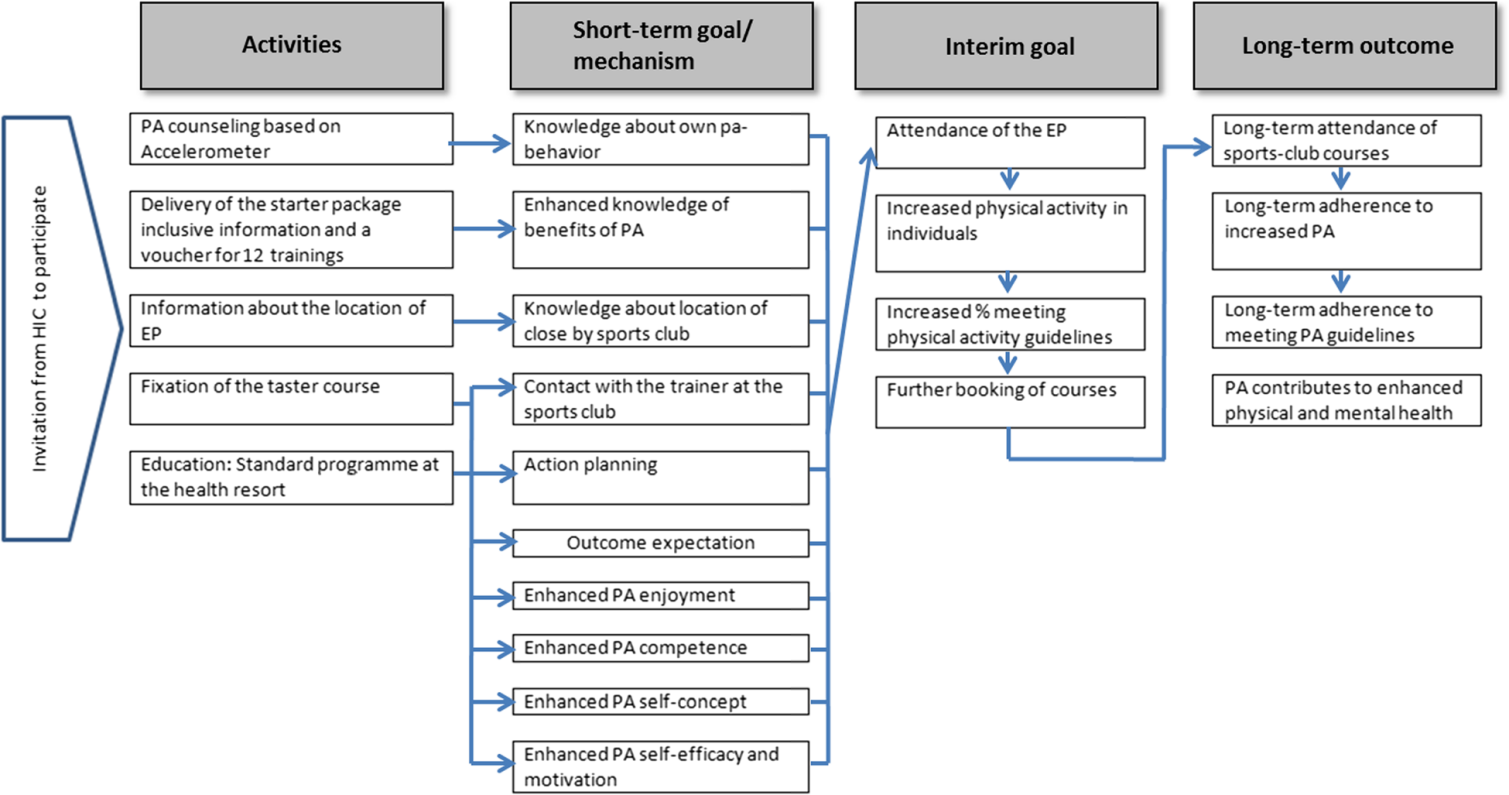
Logic model of the study

To plan the evaluation, a logic model has been developed. The model is based on the assumption that regular physical activity has a positive impact on health for those who were inactive. [19]. This long-term outcome is shown on the right-hand side of Fig. 1. In the second column from the right, we indicate the expected interim goal of the programme, i.e. participants of the intervention group who received the starter package visit the regional standardised exercise programmes after they return home. The key mechanisms (or psychological mediators) that if enhanced are likely to lead to increased attendance of the exercise programmes are shown in the second column from the left. Finally, in the far left column of Fig. 1, the activities which will take place during the residential stay are listed.

### Measurements

All study participants will be evaluated at two or three points in time (see Table 2).

**Table 2:** Time schedule for the different measurements

	Baseline	Baseline fitness^a^	Follow up1^b^	Follow up2^c^
	PA & mediators		PA & mediators	PA & mediators and fitness
Physical activity (accelerometer, log book)	✓		✓	✓
Questionnaire	✓		✓	✓
Anthropometry		✓		✓
Cardiovascular fitness (bicycle ergometer test)		✓		✓
Physical function (6MWT, handgrip, balance)		✓		✓
Blood chemistry – laboratory		✓		✓
Medication		✓		✓

^a^directly after the stay at a health resort

^b^= 10 weeks after the beginning of the stay in the health resort

^c^= 12 months after the beginning of the stay in the health resort

- Physical activity will be objectively measured using an accelerometer (GENEActiv) together with a physical activity log book before the residential stay (T1), 10 weeks after the first day at the health resort (T2) and 12 months after the first day at the heath resort (T3). Participants will be instructed to wear the accelerometer during seven complete and consecutive days, 24 h each day. At the same time, a log book should be filled in detailing walking and cycling for transport, walking and cycling during leisure time and strength training. At the same time points, participants will be asked to fill in the Office in Motion Questionnaire [20].
- All study participants (from the intervention and the control groups) will be encouraged to participate in an ergometer test, three functional fitness tests and some laboratory tests. Cardiovascular fitness will be tested with a bicycle ergometer to exhaustion [21, 22]. In general, subjects should reach maximum exercise capacity in 8 to 12 min [23]. To ensure that the ergometer test protocols can be compared, all participants will have to perform the same test protocol, which consists of a 35-watt initial loading increment for 2 min. Subsequently, the work load will be increased by 10 watts every minute. Work capacity will be measured in watts. Maximum heart rate and Borg-RPE will be documented as parameters for exhaustion [24].
- Physical function will be evaluated with the 6-Minute Walk Test [25]. Participants will be encouraged to walk a maximum possible distance within 6 min, during which modifying the speed will be tolerated. Muscle strength will be measured using a handgrip dynamometer (Jamar hydraulic hand dynamometer J00105) [26] and balance will be tested using the one-leg stand [27].
- Quality of life will be assessed by the World Health Organisation Quality of Life WHOQOL-BREF scale [28]. Psychological mediators such as motivation [29], satisfaction with life [30], satisfaction with the built environment [5], positive and negative effects [31], self-efficacy [32], social support [33] and enjoyment of physical activity (single item) will be determined via questionnaire. Country of birth and education level will also be recorded.
- As anthropometric measurements, body weight, body height and abdominal girth will be evaluated.
- Laboratory parameters, including glucose, high density lipoprotein, low density lipoprotein, triglyceride, creatinine, sodium, potassium, calcium, chloride, phosphate, bicarbonate, GGT, GOT, GPT, BNP, NT-proBNP and HbA1c, will be recorded, and medication will be derived from the patient’s chart.

### Process evaluation: patient flow

**Fig. 2:**
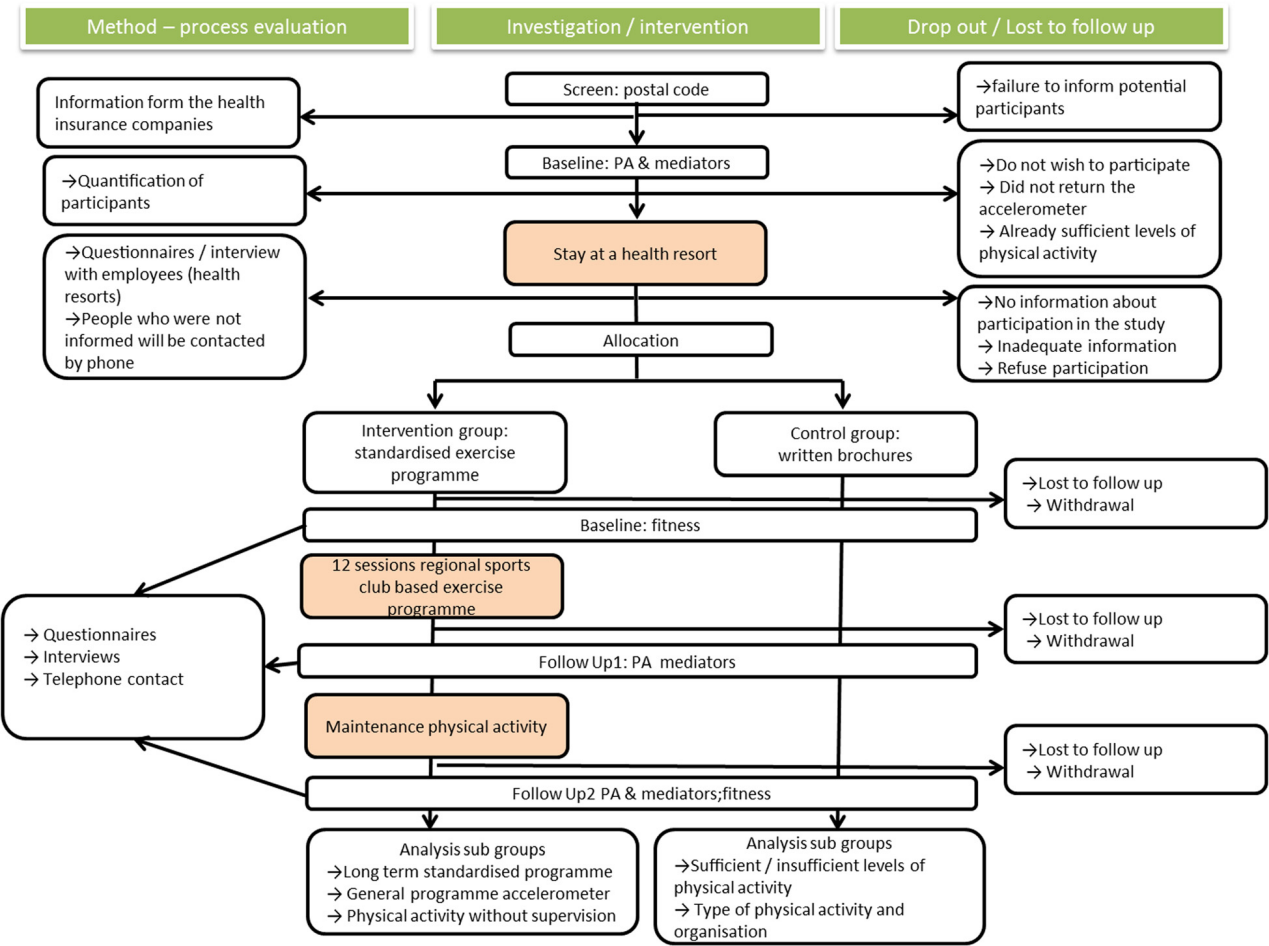
Flow chart of the study

We will analyse how many of the potential participants are willing to take part in the physical activity measurements (accelerometer together with the log book) prior to the residential stay. After that, the number of people who start the exercise programme will be evaluated, as well as the reasons for continuing the programme or quitting it after the 12 free sessions. The number of attended sessions will also be evaluated. Patient flow is shown in Fig. 2.

Within this project, the implementation of an interface function at the health resorts will be tested. Therefore, the focus will be on the processes during the residential stay, which should lead to participation in the regional exercise programmes. The key outcome will be the number/percentage of people who are informed at the health resorts and are finally allocated to the regional exercise programmes. The counterpart to the interface function in the health resorts will be the regional, standardised, sports club based exercise programmes. The process evaluation will be aimed at determining the factors that are necessary to implement new community-based health-oriented physical activity programmes. The process evaluation is summarized in Table 3.

**Table 3:** Process evaluation: questions and measures

Task	Process evaluation	Measures
Willingness for physical activity measurement	How many potential participants return the form?	Number of people who return the form
	How many are willing to participate in the activity measurement?	Number of people who are willing to participate in the activity measurement
Information and recruitment during the residential stay	How many persons are generally informed?	Number of informed people
	Which materials are used?	Number of people who arrange a start date for the initial exercise programme
	On which occasion is the regional programme presented?	
	Percentage of people for whom a start time of the regional programme has been scheduled	
Initial exercise programme	How many people actually start the regional programme?	Number of people who start the initial exercise programme
	Reasons for sustainable participation or for drop-out	Number of successful participants
Long-term exercise programme	How many people continue with the long-term programme?	Number of people who start the long-term exercise programme
	Reasons for sustainable participation or for drop-out	Number of successful participants

### Statistical analyses

All the statistical analyses will be performed with IBM® SPSS® Statistics for Windows, Version 20 (IBM Corp., Armonk, NY, U.S.). P-values <0.05 will be considered to be statistically significant, and all tests will be two-sided. Data exploration using descriptive statistical analysis and inferential statistics will be performed. The sample data will be carried out by frequencies or percentages (categorical variables), means and standard deviations (continuous variables) and graphics. T-tests and chi-square tests will be used to compare the groups. If a normal distribution is not met, non-parametric tests will be applied. Analysis of covariance (ANCOVA) (comparing parameters after the intervention and after the follow-ups) between the intervention and control groups will be adjusted for the baseline values as the covariate analysis is performed.

## Discussion

This study is aimed at evaluating the effects and processes of regional, standardised, sports club based exercise programmes, which are promoted during a residential stay at health resorts. Thus, the health sector and the sports sector need to be linked. While the health sector informs and recruits the participants, the sports sector provides the regional, standardised, exercise programmes. In many European countries, widespread networks of sports clubs exist, but they are hardly ever used for standardised health-enhancing exercise programmes. In Germany, for instance, more than 91,000 sports clubs are registered, and 29.7 % of them provide general leisure time oriented programmes [34]. These programmes primarily focus on target groups that are already somewhat active [35]. Only 4.4 % of all German sports clubs provide exercise programmes that are financially supported by health insurance companies. In England and Scotland, studies have investigated professional football clubs as settings to provide prevention programmes [36, 37]. Although these programmes have showed great effects, they might not be easily transferable to other countries. In Great Britain, football is a kind of religion, and being on the same playing fields as the professional players was extremely important for the participants [38]. Certainly, in many other countries, football does not have the same importance. Similar to the studies in Great Britain, sports clubs as a non-clinical setting have also been used in an Austrian feasibility study [10]. However, differing from this feasibility study, no bicycle ergometers for cardiovascular exercise or multi-towers for resistance training were used in the regional, standardised, exercise programme. To ensure health effects, it is important to realise regular aerobic physical activity with a moderate or vigorous intensity, as well as muscle-strengthening activities [1]. The manual with the predefined exercise sessions will help to provide diverse aerobic exercises, as well as strength training, and is in line with the different guidelines describing the access to regional structures which provide exercise programmes as an important task in physical activity promotion [8, 39].

A major strength of the study will be the fact that different sectors will be cooperating and that physical activity will be measured objectively. Another strength will the process evaluation with the objective to learn about the implementation of exercise referral in medical settings. The study intervention itself will be another strength: the regional, standardised, exercise programmes have been developed by scientists, together with representatives of sports clubs. If the study participants attend the exercise programmes twice a week, they will almost meet the WHO physical activity recommendations.

A limitation of the study might be the recruitment that will be undertaken during the stay at the health resorts. Although the procedure is described in detail, it will be undertaken by employees of the resorts, and not by the study stuff. This is an important issue, because taking sustainability into account, it is the staff in the health resorts who should encourage people to attend regional, standardised, exercise programmes after their stay at health resorts in the future. Therefore, one of the most important questions in the process evaluation is how the staff in the health resorts succeed in recruiting participants for the regional programmes. A well-known barrier for the health professionals working in the resorts is that they are confronted with numerous patients, and that only a small number of them live in communities where regional exercise programmes are provided. As was found in other lifestyle programmes, the number of drop-outs or those lost to follow-up might be a limiting factor [40]. Due to the fact that the measurement of physical activity and its feedback in the preliminary studies was found to be appealing for the study participants, the number of drop-outs will hopefully be small. One factor which may be a slight limitation to the study is that people who quit the regional exercise programme or even never start it could also reach the weekly physical activity recommendation by being physically active independent of the regional exercise programme. Data from the questionnaire and information from the trainers will help to identify these cases.

The second physical activity measurement will be quite soon (10 weeks after the first day at the health resort) after the residential stay and has been discussed at depth within the study group. Follow Up1 will only consist of physical activity measurements and the assessment of psychological mediators. The reason for this is that we are interested in whether short-term changes in physical activity patterns are possible within a short period of time. This information will also be interesting for the 12-months follow-up, because, compared to Follow Up1, the weekly amount of health-enhancing physical activity might have declined, but it should still have increased compared to the baseline level.

In summary, linking the health sector with the sports sector enables the access to community-based structures to increase the weekly amount of health-enhancing physical activity.

## Abbreviations

MVPA: Moderate- to vigorous-intensity physical activity
PA: Physical activity
